# Sequencing and Genomic Diversity Analysis of IncHI5 Plasmids

**DOI:** 10.3389/fmicb.2018.03318

**Published:** 2019-01-14

**Authors:** Quanhui Liang, Xiaoyuan Jiang, Lingfei Hu, Zhe Yin, Bo Gao, Yuee Zhao, Wenhui Yang, Huiying Yang, Yigang Tong, Weixuan Li, Lingxiao Jiang, Dongsheng Zhou

**Affiliations:** ^1^Department of Laboratory Medicine, Zhujiang Hospital, Southern Medical University, Guangzhou, China; ^2^Department of Clinical Laboratory, The First People’s Hospital of Foshan, Foshan, China; ^3^State Key Laboratory of Pathogen and Biosecurity, Beijing Institute of Microbiology and Epidemiology, Beijing, China

**Keywords:** IncHI5 plasmids, IMP, VIM, mobile elements, multidrug resistance

## Abstract

IncHI plasmids could be divided into five different subgroups IncHI1–5. In this study, the complete nucleotide sequences of seven *bla*_IMP_- or *bla*_VIM_-carrying IncHI5 plasmids from *Klebsiella pneumoniae*, *K. quasipneumoniae*, and *K. variicola* were determined and compared in detail with all the other four available sequenced IncHI5 plasmids. These plasmids carried conserved IncHI5 backbones composed of *repHI5B* and a *repFIB*-like gene (replication), *parABC* (partition), and *tra1* (conjugal transfer). Integration of a number of accessory modules, through horizontal gene transfer, at various sites of IncHI5 backbones resulted in various deletions of surrounding backbone regions and thus considerable diversification of IncHI5 backbones. Among the accessory modules were three kinds of resistance accessory modules, namely Tn*10* and two antibiotic resistance islands designated ARI-A and ARI-B. These two islands, inserted at two different fixed sites (one island was at one site and the other was at a different site) of IncHI5 backbones, were derived from the prototype Tn*3*-family transposons Tn*1696* and Tn*6535*, respectively, and could be further discriminated as various intact transposons and transposon-like structures. The ARI-A or ARI-B islands from different IncHI5 plasmids carried distinct profiles of antimicrobial resistance markers and associated mobile elements, and complex events of transposition and homologous recombination accounted for assembly of these islands. The carbapenemase genes *bla*_IMP-4_, *bla*_IMP-38_ and *bla*_VIM-1_ were identified within various class 1 integrons from ARI-A or ARI-B of the seven plasmids sequenced in this study. Data presented here would provide a deeper insight into diversification and evolution history of IncHI5 plasmids.

## Introduction

Plasmids of the H incompatibility (IncH) group show two types of surface exclusion and incompatibility interactions, namely IncHI and IncHII (Taylor and Grant, 1977). The IncHI group can be further divided into five subgroups IncHI1 to IncHI5 based on their nucleotide sequence homology (Liang et al., 2017), but the incompatibility interactions between these subgroups are still unclear because no one has actually done real incompatibility tests. IncHI plasmids, often >200 kb in size, have a wide host range including Enterobacteriaceae species and several other Gram-negative organisms (Maher and Taylor, 1993). IncHI1–5 have different replication gene profiles, namely *repHI1A*+*repHI1B*+*repFIA*-like, *repHI2A*+*repHI2C*, *repHI3B*+*repFIB*-like, *repHI4A*+*repHI4B*, and *repHI5B*+*repFIB*-like, respectively (Liang et al., 2017). IncHI plasmids generally possess two conjugal transfer regions *tra1* and *tra2*, and the ability of conjugative transfer is thermosensitive and the transfer efficiency is optimal between 22 and 30°C, but inhibited at 37°C (Sherburne et al., 2000). IncHI plasmids are important vectors of genes encoding for resistance not only to heavy metals (such as mercuric ions, copper, silver ions, tellurite, arsenate, and arsenite) but to antibiotics (such as β-lactams including carbapenems, quinolones, aminoglycosides, tetracyclines, amphenicols, and fosfomycin) (Cain and Hall, 2012).

Currently only four fully sequenced IncHI5 plasmids are available (last accessed June 28^th^, 2017), including pKOX_R1 (Accession No. CP003684) (Huang et al., 2013), pKpNDM1 (Accession No. JX515588) (Li et al., 2014), pKP04VIM (Accession No. KU318421), and pYNKP001-dfrA (Accession No. KY270853) (Liang et al., 2017). This study presents the complete nucleotide sequences of six *bla*_IMP_-carrying IncHI5 plasmids and a *bla*_VIM_-carrying one, and further comprehensive genomic comparison of all the 11 available sequenced IncHI5 plasmids enable to gain a deeper insight into genomic variation and evolution of IncHI5 plasmids.

## Materials and Methods

### Bacterial Strains

*Klebsiella quasipneumoniae* A708 and *K. pneumoniae* A324 were isolated in 2014 from the blood specimens of two different patients from a teaching hospital in Guangzhou City, China. *K. pneumoniae* 13190 and 12208, and *K. variicola* 13450 were recovered in 2013 from the sputum, sputum and blood specimens of three different patients from a teaching hospital in Hangzhou City, China, respectively. *K. pneumoniae* 11219 was isolated in 2013 from a sputum specimen of a patient from a teaching hospital in Hefei City, China. *K. pneumoniae* 19051 was recovered in 2011 from a urine specimen of a patient from a public hospital in Ningbo, China.

### Phenotypic Assays

Activity of Ambler class A/B/D carbapenemases in bacterial cell extracts was determined by a modified CarbaNP test (Wei et al., 2016). Bacterial antimicrobial susceptibility was tested by BioMérieux VITEK 2 and interpreted as per the 2017 CLSI guidelines (CLSI, 2017).

### Conjugal Transfer

Conjugal transfer experiments were carried out with the rifampin-resistant *Escherichia coli* EC600 used as a recipient and the *bla*_IMP_-positive A324 isolate as a donor. Three milliliters of overnight cultures of each of donor and recipient bacteria were mixed together, harvested and resuspended in 80 μL of Brain Heart Infusion (BHI) broth (BD Biosciences). The mixture was spotted on a 1 cm^2^ hydrophilic nylon membrane filter with a 0.45 μm pore size (Millipore) that was placed on BHI agar (BD Biosciences) plate and then incubated for mating at 22°C for 24 h. Bacteria were washed from filter membrane and spotted on Muller-Hinton (MH) agar (BD Biosciences) plates containing 2500 μg/mL rifampin together with 4 μg/mL meropenem for selecting an *E. coli* transconjugant carrying *bla*_IMP_ (pA324-IMP).

### Electroporation

To prepare competent cells for electroporation, 200 mL of overnight culture of *E. coli* TOP10 in Super Optimal Broth (SOB) at an optical density (OD_600_) of 0.4 to 0.6 was washed three times with electroporation buffer (0.5 M mannitol and 10% glycerol) and concentrated into a final volume of 2 mL. One microgram of DNA were mixed with 100 μL of competent cells for electroporation at 25 μF, 200 Ω and 2.5 Kv. The resulting cells were suspended in 500 μL of SOB and an appropriate aliquot was spotted on SOB agar plates containing 4 μg/mL meropenem for selecting of an electroporant carrying *bla*_IMP_ (pA324-IMP).

### Sequencing and Sequence Assembly

Genomic DNA was isolated from each of the A708, 13190, 11219, 12208, 13450, and 19051 isolates using a Qiagen blood & cell culture DNA maxi kit. Genome sequencing was performed with a sheared DNA library with average size of 15 kb (ranged from 10 to 20 kb) on a PacBio RSII sequencer (Pacific Biosciences, Menlo Park, CA, United States), as well as a paired-end library with an average insert size of 400 bp (ranged from 150 to 600 bp) on a HiSeq sequencer (Illumina, San Diego, CA, United States). The paired-end short Illumina reads were used to correct the long PacBio reads utilizing *proovread* (Hackl et al., 2014), and then the corrected PacBio reads were assembled *de novo* utilizing *SMARTdenovo*^1^.

Plasmid DNA was isolated from the A324-IMP-TOP10 electroporant using a Large Construct Kit (Qiagen, Germany) and then sequenced from a mate-pair library with average insert size of 5 kb (ranged from 2 to 10 kb) using a MiSeq sequencer (Illumina, San Diego, CA, United States). Quality control, removing adapters and low quality reads, were performed using *Trimmomatic* 0.36 (Bolger et al., 2014). The filtered clean reads were then assembled using *Newbler* 2.6 (Nederbragt, 2014), followed by extraction of the consensus sequence with *CLC Genomics Workbench* 3.0 (Qiagen Bioinformatics). *Gapfiller* V1.11 (Boetzer and Pirovano, 2012) was used for gap closure.

### Sequence Annotation and Comparison

Open reading frames and pseudogenes were predicted using *RAST* 2.0 (Brettin et al., 2015) combined with *BLASTP/BLASTN* searches (Boratyn et al., 2013) against the *UniProtKB/Swiss-Prot* database (Boutet et al., 2016) and the *RefSeq* database (O’Leary et al., 2016). Annotation of resistance genes, mobile elements, and other features was carried out using the online databases including *CARD* (Jia et al., 2017), *ResFinder* (Zankari et al., 2012), *ISfinder* (Siguier et al., 2006), *INTEGRALL* (Moura et al., 2009), and *Tn Number Registry* (Roberts et al., 2008). Multiple and pairwise sequence comparisons were performed using *MUSCLE* 3.8.31 (Edgar, 2004) and *BLASTN*, respectively. Gene organization diagrams were drawn in *Inkscape* 0.48.1^2^.

### Phylogenetic Analysis

The backbone regions of indicative plasmids were aligned using *MUMmer* 3.0 (Kurtz et al., 2004). Inference of homologous recombination was performed using *ClonalFrameML* (Didelot and Wilson, 2015) to remove recombination-associated single-nucleotide polymorphisms (SNPs). A maximum-likelihood tree was constructed from recombination-free SNPs using *MEGA7* (Kumar et al., 2016) with a bootstrap iteration of 1000.

### Nucleotide Sequence Accession Numbers

The complete nucleotide sequences of plasmids p11219-IMP, p12208-IMP, p13190-VIM, p13450-IMP, p19051-IMP, pA324-IMP, and pA708-IMP, and those of the A708, 12208, 13450, 11219, 13190, and 19051 chromosomes were submitted to GenBank under Accession Nos. MF344561 to MF344567, CP030171 to CP030174, CP026017, and CP022023, respectively.

## Results and Discussion

### Overview of Sequenced IncHI5 Plasmids

The seven plasmids sequenced in the present work varied in size from about 238 kb to nearly 345 kb with variation in the number of predicted ORFs from 261 to 379 (Table 1 and Supplementary Figure S1). All these plasmids belonged to the IncHI5 group, because each contained a conserved IncHI5 backbone especially including the IncHI5-type replication gene *repHI5B* and an additional *repFIB-*like gene (Liang et al., 2017). Table 1 also lists the features of the previously four sequenced IncHI5 plasmids. Further comparative genomics of all these 11 plasmids revealed that the IncHI5 backbones were interrupted by various accessory modules (defined as acquired DNA regions associated and bordered with mobile elements) inserted at different sites. In addition, while pKOX_R1 (Huang et al., 2013), the first sequenced IncHI5 plasmid, was used previously as the IncHI5 reference (Liang et al., 2017), the newly sequenced pA324-IMP seemed a more appropriate reference in this analysis because it contained the most complete IncHI5 backbone (Supplementary Figure S2).

**Table 1:** Major features of IncHI5 plasmids analyzed.

Plasmid	Accession number	Host bacterium	Total length (bp)	Total number of ORFs	Mean G+C content (%)	Length of Backbone (bp)	Mean G+C content Of backbone (%)	Accessory modules			
								Resistance			Non-resistance
								ARI-A (Tn*1696*-derived)	ARI-B (Tn*6535*-derived)	Other	
pA324-IMP	MF344566	*K. pneumoniae* A324	271,153	296	46.6	215,443	44.7	Tn*6382*	Tn*6381*	-	IS*903*, IS*Kpn8*, an IS*4*-related region, Tn*1722*, IS*5*, and IS*Kpn37*
pKpNDM1	JX515588	*Raoultella planticola* KpNDM1	277,682	314	46.9	201,856	44.7	Tn*6401*	Tn*6381*	Tn*10*	IS*Ec33*, an IS*4*-related region, and IS*5*
pKP04VIM	KU318421	*K. pneumoniae* KP04	274,659	305	46.8	212,954	44.6	Tn*6400*	+	-	IS*Ec33*, IS*Kpn28*, IS*Kox3*, IS*Kpn37*, an IS*4*-related region, and Tn*6344*
p13190-VIM	MF344563	*K. pneumoniae* 13190	288,771	322	47.3	214,781	44.7	Tn*6384*	+	-	IS*Ec33*, IS*Kpn28*, IS*Kpn37*, an IS*4*-related region, and Tn*6344*
p12208-IMP	MF344562	*K. pneumoniae* 12208	323,333	351	46.8	214,525	44.7	Tn*6383*^#^	+^#^	-	IS*903*, IS*Ec33*, IS*Ec33*:IS*10L*, IS*10L*, IS*Kpn28*, IS*Kpn21*:IS*Kpn38*, Tn*6344*, and IS*Kpn37*
p11219-IMP	MF344561	*K. pneumoniae* 11219	319,852	344	47	199,392	44.2	+^#^	+^#^	-	IS*903*, IS*Ec33*, IS*Kpn8*–IS*Kpn28*, and IS*Kpn21*
pKOX_R1	CP003684	*K. michiganensis* E718	353,865	384	47.5	214,073	44.7	+^#^	+^#^	-	IS*903*, IS*102*, IS*Ec33*, IS*Kox3*, Tn*6344*, IS*Kpn21*, and IS*Kpn28*
p13450-IMP	MF344564	*K. variicola* 13450	344,478	379	47.4	212,597	44.7	+^#^	+^#^	-	IS*903*, IS*Ec33*, IS*Kpn37*, Tn*6344*, IS*Kpn21*, and IS*Kpn28*
p19051-IMP	MF344565	*K. pneumoniae* 19051	316,843	349	48.3	172,621	45.7	+^#^	+^#^	-	IS*903*, IS*Ec33*, IS*10L*, IS*Kpn37*, Tn*6344*, IS*Kpn21*, and IS*Kpn28*
pYNKP001-dfrA	KY270853	*R. ornithinolytica* YNKP001	234,154	274	46.2	190,173	44.6	Tn*6338*	-	-	IS*Kpn28*, IS*Kpn21*, and Tn*6344*
pA708-IMP	MF344567	*K. quasipneumoniae* A708	238,703	261	47.2	171,575	44.3	+	-	-	IS*903B*, IS*Kpn28*, IS*Kox1*, and Tn*6344*


*Plasmids pKOX_R1 (Huang et al., 2013), pKpNDM1 (Li et al., 2014) and pKP04VIM were derived from GenBank, while pYNKP001-dfrA (Liang et al., 2017) and all the other seven plasmids (this study) were fully sequenced in our laboratory. A genomic comparison of all these 11 plasmids was interpreted in the main text. +: presence ARI-A or ARI-B, but identified as a transposon-like structure derived from Tn1696 or Tn6535, respectively; -: absence. ^#^: a translocation event occurred between ARI-A and ARI-B in the relevant plasmid (see Supplementary Figure S4 for detail).*

This large collection of IncHI5 plasmids allowed us to accurately distinguish backbone and accessory modules. This has allowed us to gain a deeper understanding of the evolution and diversification of IncHI5 plasmids.

### General Comparison of Backbone Sequences

**FIGURE 1:**
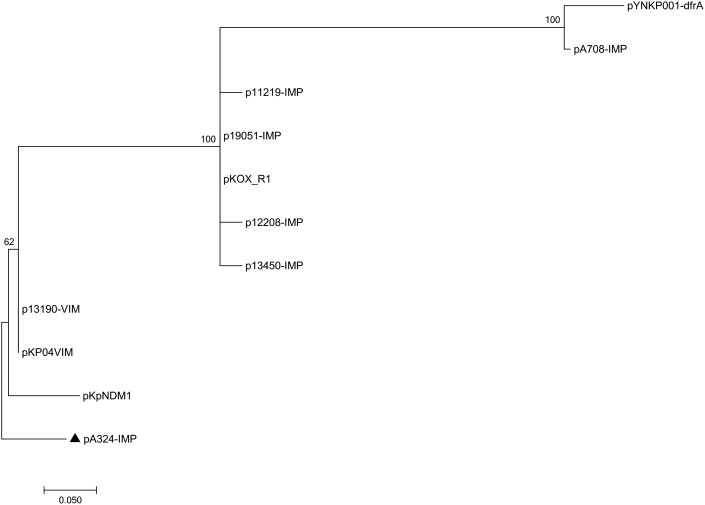
Maximum-likelihood tree. The degree of support (percentage) for each cluster of associated taxa, as determined by bootstrap analysis, is shown next to each branch. The bar corresponds to the scale of sequence divergence. The triangle indicates IncHI5 reference plasmid pA324-IMP.

Pairwise sequence comparison using *BLASTN* showed that these 11 plasmids had >99% nucleotide identity across >73% of their backbone sequences (Supplementary Table S1). The major IncHI5 backbone genes or gene loci (including *repHI5B* together with its iterons and *repFIB-*like for replication, *parABC* for partition, and *tra1* for conjugal transfer) were conserved among all these 11 plasmids. Two conjugal transfer regions *tra1* and *tra2* were found in IncHI5 plasmids, but some of these plasmids lost *tra2*, which would impair their self-transferability (Supplementary Table S3). A total of 574 core SNPs (among them 115 were recombination-free) were identified from the backbone regions of these 11 plasmids. A maximum likelihood phylogenetic tree was constructed using these 115 recombination-free SNPs, and accordingly these 11 plasmids could be assigned into three clades I, II, and III (Figure 1).

### Classification of Accessory Modules

These 11 plasmids harbored different profiles of accessory modules (Table 1) and different collections of resistance genes (Supplementary Table S2). The accessory modules were further divided into resistance (containing resistance genes) and non-resistance (containing no resistance genes) ones (Table 1). The resistance accessory modules included Tn*10* (an IS*10*-composite transposon carrying class B tetracycline-resistance genes) and two antibiotic resistance islands designated ARI-A and ARI-B. The presence and modular organization of ARI-A and ARI-B islands in the seven newly sequence plasmids and pYNKP001-dfrA (Liang et al., 2017) were validated by a set of PCR amplifications (see Figures 3, 4 for location of PCR primers and expected amplicons) that targeted various key jointing fragments of these islands and their surrounding backbone regions, using the genomic DNA of each corresponding wild-type isolate as template. The non-resistance accessory modules were composed of 12 different insertion sequences (ISs), four distinct IS-related regions, and two cryptic transposons Tn*6344* (Supplementary Figure S3) and Tn*1722*.

### Massive Gene Acquisition and Loss in IncHI5 Plasmids

**FIGURE 2:**
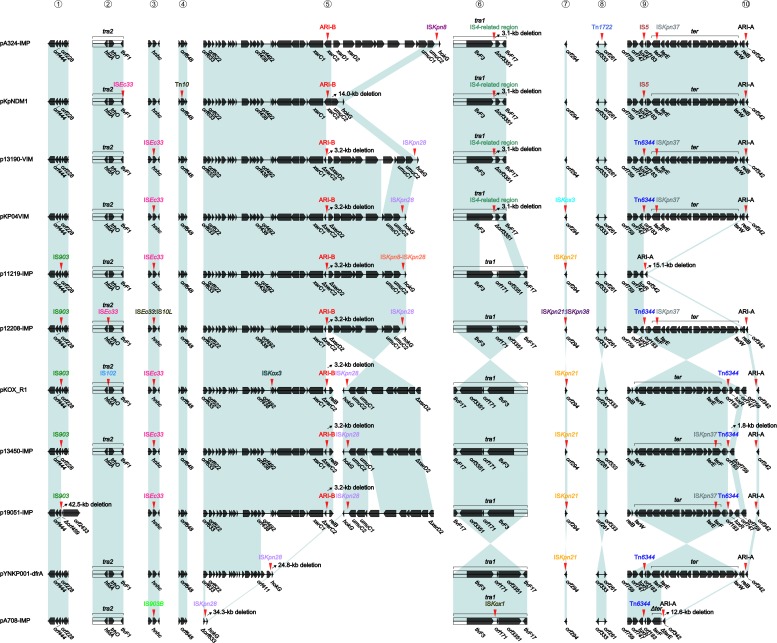
Sites of insertion of accessory modules. Genes are denoted by arrows. Genes, mobile elements and other features are colored based on function classification. Shading denotes regions of homology (>95% nucleotide identity).

At least 10 major events of gene acquisition/loss accounted for modular diversity of these 11 plasmids across their genomes (Figure 2). First, IS*903* was inserted at a site upstream of *orf444* in five plasmids, additionally resulting in a 42.5-kb deletion (containing the whole *tra2* region) in p19051-IMP. Second, IS*Ec33*, IS*Ec33* and IS*102* were inserted at different sites within the *tra2* regions of pKpNDM1, p12208-IMP and pKOX_R1, respectively. Third, IS*903B* in pA708-IMP, IS*Ec33* in six plasmids, and IS*Ec33*:IS*10L* in p12208-IMP were inserted within *hnhc* (HNH endonuclease), splitting it into two separate parts Δ*hnhc*-5′ and Δ*hnhc*-3′. Fourth, in pKpNDM1, Tn*10* was inserted at a site within *orf648*, splitting it into two separate parts Δ*orf648*-5′ and Δ*orf648*-3′. Fifth, compared to the backbone region from *orf633* to *hokG* in pA324-IMP as a prototype structure, various insertions occurred in all the other plasmids: (i) insertion of IS*Kpn28* upstream of *hokG* resulted in deletion of a 34.3-kb region in pA708-IMP and that of a 24.8-kb region in pYNKP001-dfrA, respectively; however, upstream-of-*hokG* insertion of IS*Kpn8* in pA324-IMP, that of IS*Kpn28* in six plasmids, and that of IS*Kpn8*–IS*Kpn28* in p11219-IMP did not cause deletions; and (ii) insertion of ARI-B at a site within *xerC2* occurred in the following nine plasmids, which led to a 3.2-kb deletion in seven plasmids, a 14.0-kb deletion in pKpNDM1, and no deletion in pA324-IMP. Sixth, six plasmids had complete *tra1* regions; by contrast, four additional plasmids had undergone insertion of an IS*4*-related region at a site downstream of *tivF3* (resulting in a 3.1-kb deletion), and IS*kox1* was inserted at a site between *tivF3* and *orf171* in pA708-IMP (no further deletion occurred). All the above insertions and/or deletions within *tra1* and *tra2* might impair self-transferability of corresponding plasmids. Seventh, IS*Kpn21* in five plasmids, IS*Kox3* in pKP04VIM, and IS*Kpn21*:IS*Kpn38* in p12208-IMP were inserted at a site downstream of *orf294*. Eighth, Tn*1722* was inserted at a site between *orf333* and *orf261* in pA324-IMP. Ninth, IS*5* or Tn*6344* was inserted at a site between *luxR* and *orf183* in all the night plasmids except for p11219-IMP and p13450-IMP, and additionally the Tn*6344* insertion resulted in a 1.8-kb deletion in p13450-IMP. Tenth, the ARI-A islands were inserted at a site downstream of *orf342* in all the 11 plasmids, which resulted in the 12.6-kb deletion (covering *terE*-5′ and *terABCDZW*) in pA708-IMP and the 15.1-kb deletion (covering the complete *ter* gene cluster) in p11219-IMP; additionally, IS*Kpn37* was inserted at a site within *terF* in six plasmids. The above insertions would impair tellurium resistance gene expression.

In conclusion, massive gene acquisition and loss were found in IncHI5 plasmids: a wealth of accessory modules were integrated at various sites of IncHI5 backbones, which resulted in various deletions of surrounding backbone regions and thus considerable diversification of IncHI5 backbones.

### Tn*1696*-Related ARI-A Islands

**FIGURE 3:**
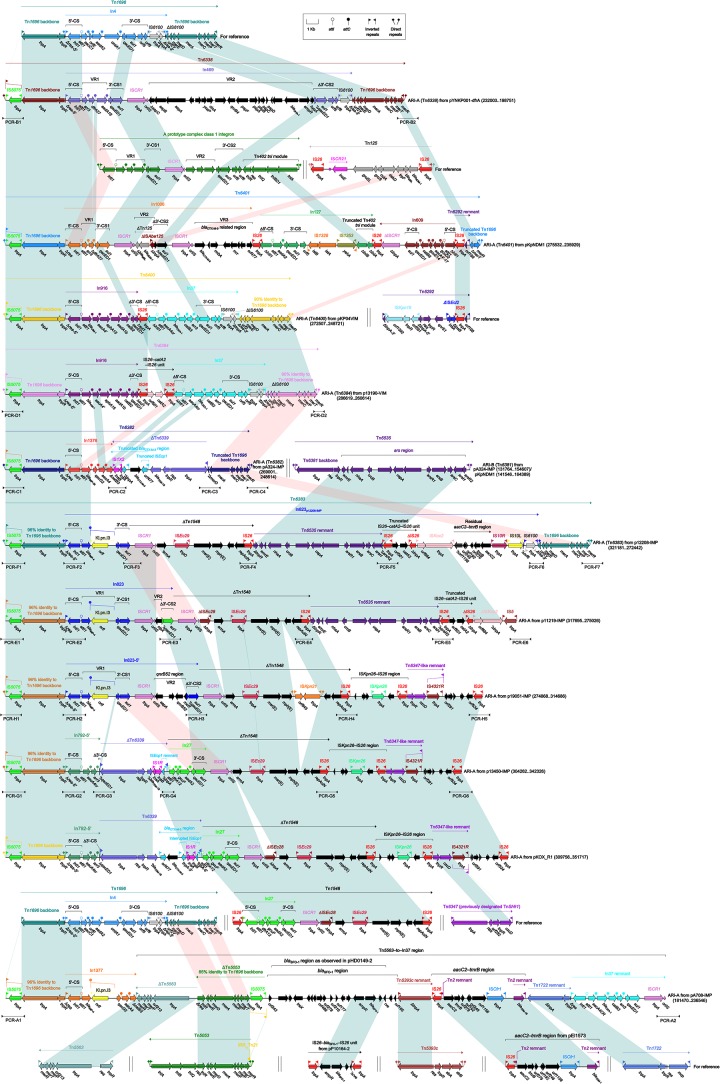
Organization of ARI-A islands and comparison to related regions. Genes are denoted by arrows. Genes, mobile elements and other features are colored based on their functional classification. Shading denotes regions of homology (nucleotide identity > 95%). Numbers in brackets indicate nucleotide positions within corresponding plasmids. The accession numbers of Tn*1696* (Partridge et al., 2001), Tn*125* (Poirel et al., 2012), Tn*6292* (Wei et al., 2016), Tn*1548* (Galimand et al., 2005), Tn*6347*, Tn*5563* (Yeo et al., 1998), Tn*5053* (Kholodii et al., 1993), IS*26*–*bla*_SFO–1_–IS*26* unit (Sun et al., 2016), Tn*5393c* (L’Abée-Lund and Sørum, 2000), *aacC2*–*tmrB* region (Partridge et al., 2012), and Tn*1722* (Allmeier et al., 1992) for reference are U12338, JN872328, KU886034, AF550415, CP000447, U88088, L40585, KX710093, AF262622, JX101693, and X61367, respectively. Arrowheads indicated location of PCR primers and expected amplicons.

The ARI-A islands (Figure 3) were found in all the 11 plasmids analyzed and identified as Tn*1696* derivatives. Tn*1696*, a unit transposon belonging to the Tn*21* subgroup of Tn*3* family, was generated from insertion of a class 1 integron In4 into the resolution (*res*) site of a primary backbone structure: IRL (inverted repeat left)–*tnpA* (transposase)–*tnpR* (resolvase)–*res*–*mer* (mercury resistance locus)–IRR (inverted repeat right) (Partridge et al., 2001).

Being similar to Tn*1696*, the ARI-A islands from six plasmids had paired terminal 38-bp IRL/IRR and were further bracketed by 5-bp direct repeats (DRs; target site duplication signals for transposition), and thus they were identified as unit transposons designated Tn*6338*, Tn*6401*, Tn*6400*, Tn*6384*, Tn*6382*, and Tn*6383*, respectively. The remaining five ARI-A islands carried only IRLs (interrupted by IS*5075* that was a hunter of terminal IRL/IRR of Tn*21* subgroup transposons (Partridge and Hall, 2003)) but did not harbor IRRs (due to truncation at 3′-terminal regions of these islands), and thus they were identified as transposon-like structures rather than intact transposons.

In conclusion, the ARI-A islands was inserted at a site downstream of *orf342* in all 11 plasmids, and further discriminated as six intact transposons (among them Tn*6384*, Tn*6382*, and Tn*6383* were novel) and five transposon-like structures. These 11 islands were derived from Tn*1696* but differed from it mainly by insertion of distinct integrons or integron-related regions instead of In4 in Tn*1696*. These integrons could be divided into concise integrons each containing a single gene cassette (GC) array, and complex integrons each harboring one or more variable regions (VRs) in addition to the GC array. These GCs and VRs commonly carried antibiotic resistance genes.

### Tn*6535*-Related ARI-B Islands

**FIGURE 4:**
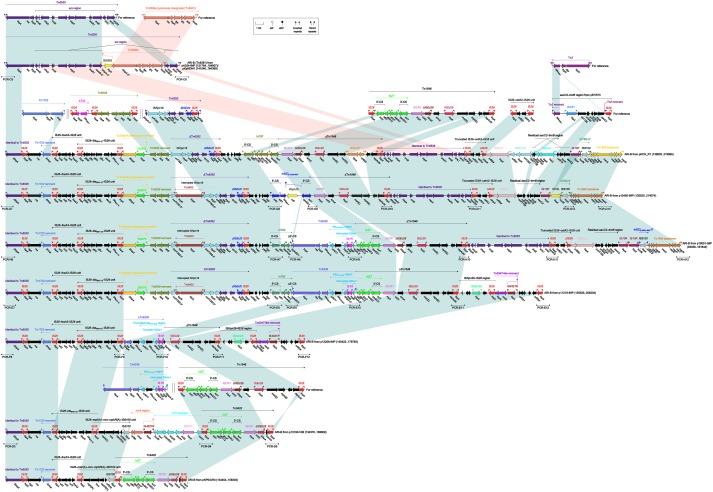
Organization of ARI-B islands and comparison to related regions. Genes are denoted by arrows. Genes, mobile elements and other features are colored based on their functional classification. Shading denotes regions of homology (nucleotide identity > 95%). Numbers in brackets indicate nucleotide positions within corresponding plasmids. The accession numbers of Tn*6535* (Tan et al., 2014), Tn*6399a* (Murata et al., 2002), Tn*1722* (Allmeier et al., 1992), Tn*6029* (Cain et al., 2010), Tn*6292* (Wei et al., 2016), Tn*1548* (Galimand et al., 2005), IS*26*–*catA2*–IS*26* unit (Xiang et al., 2015), Tn*2* (Bailey et al., 2011), *aacC2*–*tmrB* region (Partridge et al., 2012), and Tn*6339* (Liang et al., 2017) for reference are CP009706, AP004237, X61367, HQ840942, KU886034, AF550415, KY270851, HM749967, JX101693, and CP003684, respectively. Arrowheads indicated location of PCR primers and expected amplicons.

The ARI-B islands (Figure 4) as found in nine plasmids were identified as the derivatives of a prototype arsenic-resistance (*ars*) unit transposon Tn*6535*. As observed in the chromosome (Accession No. CP009706) of *Hafnia alvei* FB1 (Tan et al., 2014), Tn*6535* was assembled from integration of an *ars* region with a Tn*3*-family core transposition module *tnpA*–*res*–*tnpR*.

The ARI-B island from pA324-IMP or pKpNDM1 was identified as an unit transposon designated Tn*6381*, with presence of paired terminal 38-bp IRL/IRR and 5-bp DRs. Tn*6381* differed from Tn*6535* by insertion of a Tn*3*-family unit transposon Tn*6399b* at a site within *arsB.* Tn*6399a* (it was previously designated Tn*6901* because it was 6901 bp in length) was found in plasmid Rts1 from *Proteus vulgaris* (Murata et al., 2002) and carried several alcohol-metabolism genes (Chen et al., 2013), while interruption of *tnpA* by IS*1618* insertion turned Tn*6399a* into Tn*6399b*. Compared to Tn*6535* and Tn*6381*, all the other ARI-B islands contained only IRLs but not IRRs and identified as transposon-like structures. Various types of insertion events occurred within these ARI-B islands, leading to truncation of prototype regions as found in Tn*6535* and Tn*6381* as well as integration of foreign resistance markers and associated mobile elements.

ARI-B_pKOX_R1_ contained at least 10 resistance loci, including IS*26*–*fosA3*–IS*26* unit, IS*26*–*bla*_SHV -12_–IS*26* unit, a 3-kb Tn*6029* remnant, ΔTn*6292*, In797, ΔTn*1548*, ΔTn*6535*, a truncated IS*26*–*catA2*–IS*26* unit, a residual *aacC2*–*tmrB* region, and a *mer* region (Liang et al., 2017). The resistance markers from ARI-B_p13450-IMP_ and ARI-B_p19051-IMP_ differed from pKOX_R1 by replacement of In797 with In823 and In792, respectively; moreover, Tn*6339* (containing *bla*_TEM-1B_ and *bla*_CTX-M-3_) was inserted at a site between In792 and ΔTn*1548* in p19051-IMP. The resistance markers from ARI-B_p11219-IMP_ were composed of IS*26*–*fosA3*–IS*26*, IS*26*–*bla*_SHV -12_–IS*26*, a Tn*6029* remnant, ΔTn*6292*, In792, Tn*6339*, and ΔTn*1548.* Similarly, IS*26*–*fosA3*–IS*26*, IS*26*–*bla*_SHV -12_–IS*26*, ΔTn*6339*, and ΔTn*1548* were present in ARI-B_p12208-IMP_. The resistance markers from ARI-B_p13190-VIM_ consisted of IS*26*–*bla*_SHV -12_–IS*26*, IS*26*–*mph(A)*–*mrx*–*mphR(A)*–IS*6100*, *chrA* region, a In37 remnant and Tn*6402*, while those from ARI-B_pKP04VIM_ were composed of IS*26*–*fosA3*–IS*26*, IS*26*–*mph(A)*–*mrx*–*mphR(A)*–IS*6100* and Tn*6402.* Tn*6402* was an IS*26*-composite transposon (delimited by 4-bp DRs at both ends) derived from Tn*1548*. Tn*6402* differed from Tn*1548* by deletion of *arsB*-3′–IS*Ec29*–*msr(E)*–*mph(E)*–*repAciN* and inversion of the 3′-end copy of IS*26*.

In conclusion, the ARI-B islands, integrated at a site within *xerC2* in nine plasmids, could be further identified as Tn*6381* and eight transposon-like structures. These nine ARI-B islands were derived from Tn*6535* but differed from it by insertion of various collections of mobile elements and associated resistance genes into the original Tn*6381* backbone.

All the transposon-like structures of ARI-A or ARI-B could not be annotated as intact transposons because they lacked paired terminal inverted repeats, and complex transposition and homologous recombination events accounted for assembly and diversification of these transposons and transposon-like structures. The ARI-A or ARI-B islands from different IncHI5 plasmids carried distinct profiles of resistance markers and associated mobile elements, promoting accumulation and spread of antimicrobial resistance among bacterial species.

### Translocation of Large Regions Across ARI-A and ARI-B

Compared to the intact transposons Tn*6338*, Tn*6401*, Tn*6400*, Tn*6384*, Tn*6382*, and Tn*6383* (corresponding to ARI-A), and Tn*6381* (corresponding to ARI-B), two kinds of translocation events across ARI-A and ARI-B occurred in each of the following five plasmids (Supplementary Figure S4): (i) exchange of the ΔTn*1548*–to–IS*26* region (finally observed in ARI-B) and the ΔTn*1548*–to–Tn*6535* region (finally observed in ARI-A) in each of p12208-IMP and p11219-IMP; and (ii) movement of the Δ*orf6*–*mer* region from ARI-A to ARI-B in each of pKOX_R1, p13450-IMP, and p19051-IMP. These two kinds of translocation might be mediated by the common regions ΔTn*1548* and IS*6100*, respectively.

### Carbapenemase Genes and Related Integrons

As for the seven plasmids sequenced in this study, the carbapenemase genes *bla*_IMP-4_, *bla*_IMP-38_, and *bla*_VIM-1_ were identified within In823_p11219-IMP/p12208-IMP/p19051-IMP_, or In1377_pA708-IMP_, In1376_pA324-IMP_, and In916_p13190-VIM_ respectively, from the ARI-A islands, while a *bla*_IMP-4_ gene was found within In823_p13450-IMP_ from the ARI-B island. Of all the integrons identified in these seven plasmids, In1376 and In1377 were novel.

### Transferability and Antimicrobial Susceptibility

As a representative IncHI5 plasmid, pA324-IMP could be transferred from the A324 isolate into *E. coli* EC600 and TOP10 through conjugation and electroporation, respectively, generating the A324-IMP-EC600 transconjugant and the A324-IMP-TOP10 electroporant, respectively. This was consistent with the presence of two complete sets of *tra1* and *tra2* genes in pA324-IMP, making it self-transferable. All the above three strains had class B carbapenemase activity (data not shown), and were resistant to all the cephalosporins and carbapenems tested (Table 2), which were resulted from production of IMP or VIM enzymes in these strains.

**Table 2:** Antimicrobial drug susceptibility profiles.

Antibiotics	MIC (mg/L)/antimicrobial susceptibility				
	A324	A324-IMP-EC600	A324-IMP-TOP10	EC600	TOP10
Ampicillin	≥32/R	≥32/R	≥32/R	16/I	4/S
Ampicillin/sulbactam	≥32/R	≥32/R	≥32/R	8/S	4/S
Cefazolin	≥64/R	≥64/R	≥64/R	≤4/S	≤4/S
Ceftazidime	≥64/R	≥64/R	≥64/R	≤1/S	≤1/S
Ceftriaxone	≥64/R	≥64/R	≥64/R	≤1/S	≤1/S
Cefepime	≥64/R	≥64/R	≥64/R	≤1/S	≤1/S
Aztreonam	≥64/R	≥64/R	≥64/R	≤1/S	≤1/S
Imipenem	4/R	4/R	4/R	≤1/S	≤1/S
Meropenem	≥16/R	≥16/R	≥16/R	≤0.25/S	≤0.25/S
Amikacin	≤2/S	≤2/S	≤2/S	≤2/S	≤2/S
Tobramycin	8/I	4/S	8/I	≤1/S	≤1/S
Ciprofloxacin	≤0.25/S	≤0.25/S	≤0.25/S	≤0.25/S	≤0.25/S
Levofloxacin	≤0.25/S	0.5/S	≤0.25/S	0.5/S	≤0.25/S
Trimethoprim/sulfamethoxazole	≤20/S	≤20/S	≤20/S	≤20/S	≤20/S


*S, sensitive; R, resistant; I, intermediately resistant.*

## Author Contributions

DZ and LJ conceived the study and designed experimental procedures. QL, XJ, LH, ZY, and WY performed the experiments. QL, XJ, BG, YZ, and HY analyzed the data. QL, XJ, YT, and WL contributed reagents and materials. DZ, QL, XJ, and LJ wrote the manuscript.

## Conflict of Interest Statement

The authors declare that the research was conducted in the absence of any commercial or financial relationships that could be construed as a potential conflict of interest.

## Footnotes

1 https://github.com/ruanjue/smartdenovo

2 https://inkscape.org/en/

## Supplementary Material

FIGURE S1 Schematic maps of sequenced plasmids. Genes are denoted by arrows, and the backbone and accessory module regions are highlighted in black and color, respectively. The innermost circle presents GC-skew [(G-C)/(G+C)], with a window size of 500 bp and a step size of 20 bp. The next-to-innermost circle presents GC content. The accession numbers of pYNKP001-dfrA (Liang et al., 2017), pKP04VIM, pKpNDM1 (Li et al., 2014), and pKOX_R1 (Huang et al., 2013) for reference are KY270853, KU318421, JX515588, and CP003684, respectively.

FIGURE S2 Linear comparison of plasmid genome sequences. Genes are denoted by arrows. Genes, mobile elements and other features are colored based on function classification. Shading denotes regions of homology (>95% nucleotide identity). The accession numbers of pYNKP001-dfrA (Liang et al., 2017), pKP04VIM, pKpNDM1 (Li et al., 2014), and pKOX_R1 (Huang et al., 2013) for reference are KY270853, KU318421, JX515588, and CP003684, respectively.

FIGURE S3 Organization of Tn*6344* and comparison to related region. Genes are denoted by arrows. Genes, mobile elements and other features are colored based on their functional classification. Shading denotes regions of homology (nucleotide identity > 95%).

FIGURE S4 Translocation between ARI-A and ARI-B islands. Genes are denoted by arrows. Genes, mobile elements and other features are colored based on their functional classification. Arrowheads indicated location of PCR primers and expected amplicons.

TABLE S1 **(a)** Pairwise comparison of IncHI5 sequences using BLASTN. **(b)** Pairwise comparison of plamid backbone sequences using BLASTN.

TABLE S2 Drug resistance genes in sequenced IncHI5 plasmids.

TABLE S3 Conjugation transfer features of IncHI5 plasmids analyzed.
