# Supplementary figures and images for: Sequencing and Genomic Diversity Analysis of IncHI5 Plasmids
Source: Front Microbiol. 2019 Jan 14;9:3318. doi: 10.3389/fmicb.2018.03318 (PMC6339943; doi:10.3389/fmicb.2018.03318)

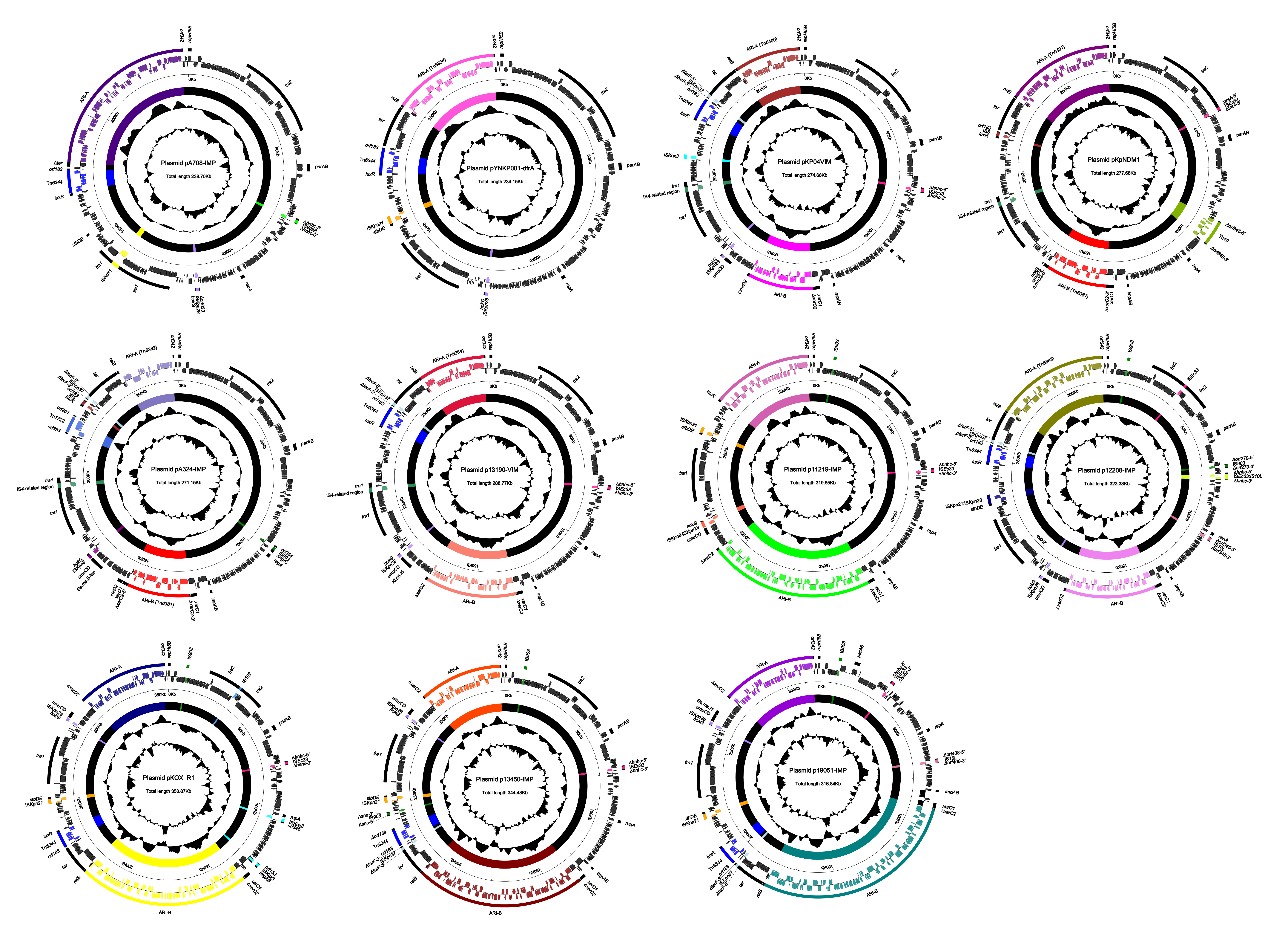

Supplement: FIGURE S1 — Schematic maps of sequenced plasmids. Genes are denoted by arrows, and the backbone and accessory module regions are highlighted in black and color, respectively. The innermost circle presents GC-skew [(G-C)/(G+C)], with a window size of 500 bp and a step size of 20 bp. The next-to-innermost circle presents GC content. The accession numbers of pYNKP001-dfrA (Liang et al., 2017), pKP04VIM, pKpNDM1 (Li et al., 2014), and pKOX_R1 (Huang et al., 2013) for reference are KY270853, KU318421, JX515588, and CP003684, respectively. [file Image_1.TIF]

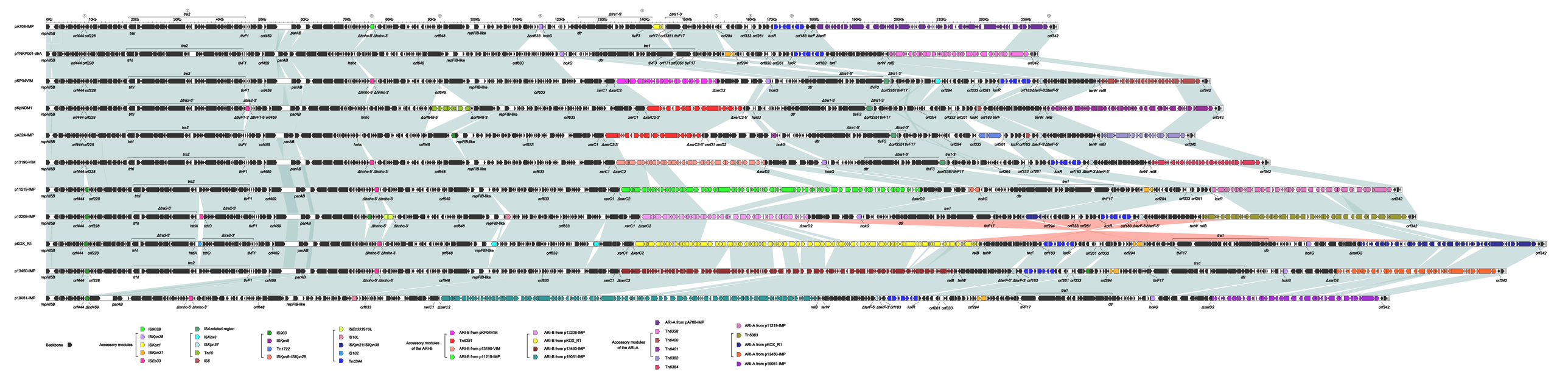

Supplement: FIGURE S2 — Linear comparison of plasmid genome sequences. Genes are denoted by arrows. Genes, mobile elements and other features are colored based on function classification. Shading denotes regions of homology (>95% nucleotide identity). The accession numbers of pYNKP001-dfrA (Liang et al., 2017), pKP04VIM, pKpNDM1 (Li et al., 2014), and pKOX_R1 (Huang et al., 2013) for reference are KY270853, KU318421, JX515588, and CP003684, respectively. [file Image_2.TIF]

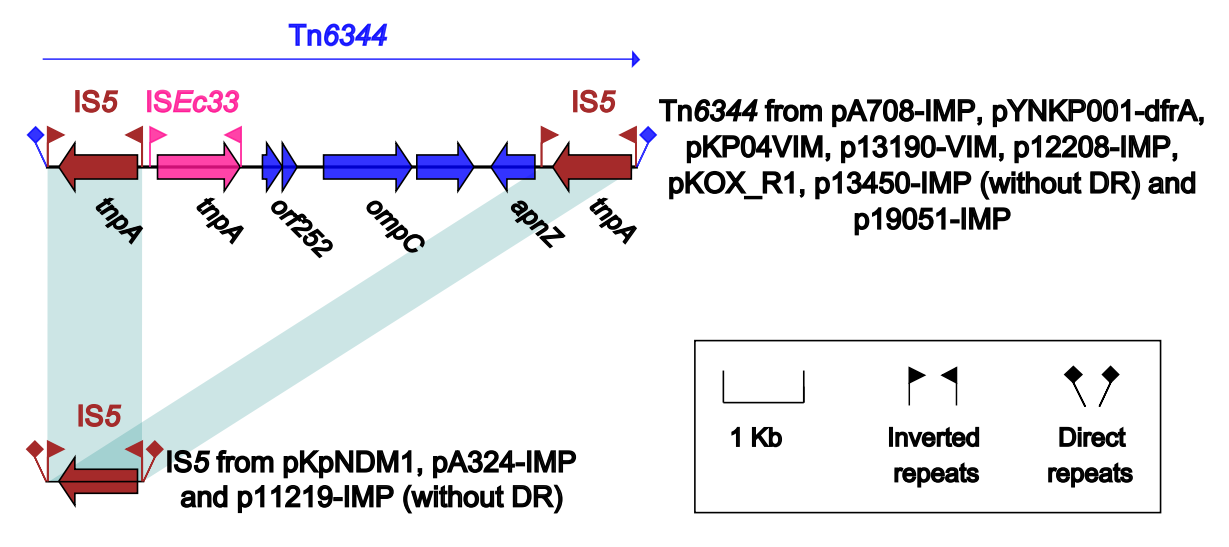

Supplement: FIGURE S3 — Organization of Tn6344 and comparison to related region. Genes are denoted by arrows. Genes, mobile elements and other features are colored based on their functional classification. Shading denotes regions of homology (nucleotide identity > 95%). [file Image_3.TIF]

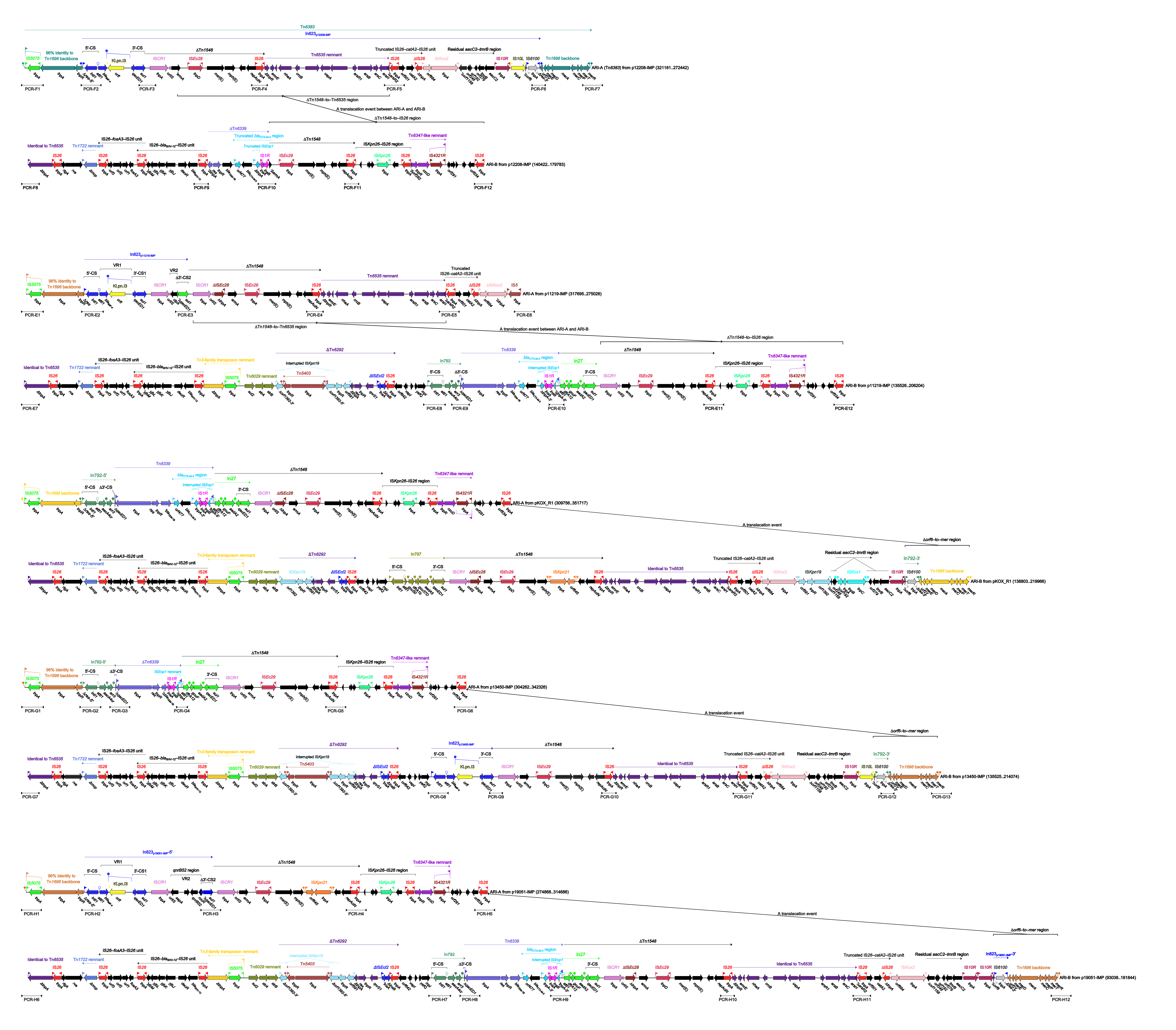

Supplement: FIGURE S4 — Translocation between ARI-A and ARI-B islands. Genes are denoted by arrows. Genes, mobile elements and other features are colored based on their functional classification. Arrowheads indicated location of PCR primers and expected amplicons. [file Image_4.TIF]
