# Supplementary material for: Sequencing and Genomic Diversity Analysis of IncHI5 Plasmids
Source: Front Microbiol. 2019 Jan 14;9:3318. doi: 10.3389/fmicb.2018.03318 (PMC6339943; doi:10.3389/fmicb.2018.03318)
Supplement: TABLE S2 — Drug resistance genes in sequenced IncHI5 plasmids. [file Table_2.docx]

**Table S2|****Drug resistance genes in sequenced IncHI5 plasmids**

| **Plasmid** | **Resistance marker** | **Resistance phenotype** | **Nucleotide**  **position** | **Region located** |
| --- | --- | --- | --- | --- |
| pA708-IMP | *ter* locus | Tellurium resistance | 180109..181470 | Plasmid backbone |
|  | *sul1* | Sulphonamide resistance | 183648..184487 | ARI-A |
|  | *qacED1* | Quaternary ammonium  compound resistance | 184481..184828 |  |
|  | *arr3* | Rifampicin resistance | 185051..185503 |  |
|  | *catB3* | Phenicol resistance | 185588..186220 |  |
|  | *bla*_OXA-1_ | Beta-lactam resistance | 186358..187188 |  |
|  | *aacA4CR* | Fluoroquinolone and aminoglycoside resistance | 187319..187918 |  |
|  | *bla*_TEM-1B_ | Beta-lactam resistance | 193292..194152 |  |
|  | *tmrB* | Tunicamycin resistance | 198569..199111 |  |
|  | *aacC2* | Aminoglycoside resistance | 199124..199984 |  |
|  | *bla*_SFO-1_ | Beta-lactam resistance | 205649..206536 |  |
|  | *mer*-1 locus | Mercuric resistance | 216189..219842 |  |
|  | *mer*-2 locus | Mercuric resistance | 224486..225597 |  |
|  | Δ*catB3* | Phenicol resistance | 225652..226158 |  |
|  | *aacA4* | Aminoglycoside resistance | 226253..226807 |  |
|  | *qacG2* | Quaternary ammonium  compound resistance | 226934..227266 |  |
|  | *bla*_IMP-4_ | Beta-lactam resistance | 229445..230185 |  |
| pYNKP001-dfrA | *ter* locus | Tellurium resistance | 184814..197752 | Plasmid backbone |
|  | *mer* locus | Mercuric resistance | 198789..202765 | ARI-A  (Tn*6338*) |
|  | *sul1* | Sulphonamide resistance | 205180..206019 |  |
|  | *∆qacED1* | Quaternary ammonium  compound resistance | 206013..206289 |  |
|  | *bla*_DHA-1_ | Beta-lactam resistance | 207596..208735 |  |
|  | *qnrB4* | Quinolone resistance | 212855..213502 |  |
|  | *sul1* | Sulphonamide resistance | 222233..223072 |  |
|  | *qacED1* | Quaternary ammonium  compound resistance | 223066..223413 |  |
|  | *aadA16* | Aminoglycoside resistance | 223530..224375 |  |
|  | *dfrA27* | Trimethoprim resistance | 224556..225029 |  |
|  | *arr3* | Rifampicin resistance | 225162..225614 |  |
| pKP04VIM | *fosA3* | Fosfomycin resistance | 143130..143546 | ARI-B |
|  | *mph*(A) | Macrolide resistance | 144758..145663 |  |
|  | *dfrA12* | Trimethoprim resistance | 151054..151551 |  |
|  | *aadA2* | Aminoglycoside resistance | 151959..152750 |  |
|  | *qacED1* | Quaternary ammonium  compound resistance | 152914..153261 |  |
|  | *sul1* | Sulphonamide resistance | 153255..154094 |  |
|  | *ter* locus | Tellurium resistance | 233528..247722 | Plasmid backbone |
|  | *mer* locus | Mercuric resistance | 248759..252714 | ARI-A  (Tn*6400*) |
|  | *sul1* | Sulphonamide resistance | 255450..256289 |  |
|  | *qacED1* | Quaternary ammonium  compound resistance | 256283..256630 |  |
|  | *arr3* | Rifampicin resistance | 256853..257305 |  |
|  | *catB3* | Phenicol resistance | 257390..258022 |  |
|  | *bla*_OXA-1_ | Beta-lactam resistance | 258160..258990 |  |
|  | *aacA4CR* | Fluoroquinolone and aminoglycoside resistance | 259121..259720 |  |
|  | *∆sul1* | Sulphonamide resistance | 261362..261694 |  |
|  | *qacED1* | Quaternary ammonium  compound resistance | 261688..262035 |  |
|  | *catB2* | Phenicol resistance | 262204..262836 |  |
|  | *aadA1b* | Aminoglycoside resistance | 262889..263680 |  |
|  | *aphA15* | Aminoglycoside resistance | 263797..264591 |  |
|  | *aacA4'* | Fluoroquinolone and aminoglycoside resistance | 264662..265216 |  |
|  | *bla*_VIM-1_ | Beta-lactam resistance | 265324..261224 |  |
| pKpNDM1 | *tetA*(B) | Tetracycline resistance | 93448..94653 | Tn*10* |
|  | *ars* locus | Arsenic resistance | 162583..164120 | ARI-B  (Tn*6381*) |
|  | *glo* | Glyoxalase/Bleomycin resistance | 160292..160690 |  |
|  | *ter* locus | Tellurium resistance | 221992..234930 | Plasmid backbone |
|  | *mer* locus | Mercuric resistance | 235967..236790 | ARI-A  (Tn*6401*) |
|  | *aacA4'-17* | Fluoroquinolone and aminoglycoside resistance | 239295..239849 |  |
|  | *catB8* | Phenicol resistance | 240261..240893 |  |
|  | *qacED1* | Quaternary ammonium  compound resistance | 241050..241397 |  |
|  | *sul1* | Sulphonamide resistance | 241391..242230 |  |
|  | *sul1* | Sulphonamide resistance | 251069..251908 |  |
|  | *qacED1* | Quaternary ammonium  compound resistance | 251902..252249 |  |
|  | *aadA2* | Aminoglycoside resistance | 252413..253192 |  |
|  | *bla*_CTX-M-9_ | Beta-lactam resistance | 258879..259730 |  |
|  | *sul1* | Sulphonamide resistance | 262027..262866 |  |
|  | *∆qacED1* | Quaternary ammonium  compound resistance | 262860..263102 |  |
|  | *bla*_NDM-1_ | Beta-lactam resistance | 263564..264376 |  |
|  | *∆ble*_MBL_ | Bleomycin resistance | 234380..264715 |  |
|  | *sul1* | Sulphonamide resistance | 266839..267678 |  |
|  | *qacED1* | Quaternary ammonium  compound resistance | 267672..268019 |  |
|  | *∆dfrA27* | Trimethoprim resistance | 268133..268558 |  |
|  | *arr3* | Rifampicin resistance | 268691..269143 |  |
| pA324-IMP | *ars* locus | Arsenic resistance | 152801..154338 | ARI-B  (Tn*6381*) |
|  | *glo* | Glyoxalase/Bleomycin resistance | 150510..150908 |  |
|  | *ter* locus | Tellurium resistance | 233421..247615 | Plasmid backbone |
|  | *mer* locus | Mercuric resistance | 248652..252155 | ARI-A  (Tn*6382*) |
|  | *bla*_TEM-1B_ | Beta-lactam resistance | 256061..256921 |  |
|  | *bla*_CTX-M-3_ | Beta-lactam resistance | 257703..258578 |  |
|  | Δ*catB3* | Phenicol resistance | 260025..260552 |  |
|  | *aacA4* | Aminoglycoside resistance | 260647..261201 |  |
|  | *qacG2* | Quaternary ammonium  compound resistance | 261328..261660 |  |
|  | *bla*_IMP-38_ | Beta-lactam resistance | 261892..262632 |  |
| p13190-VIM | *bla*_SHV-12_ | Beta-lactam resistance | 144815..145675 | ARI-B |
|  | *mph(A)* | Macrolide resistance | 146638..147543 |  |
|  | *chrA* | Chromate resistance | 151162..152367 |  |
|  | *sul1* | Sulphonamide resistance | 154181..155020 |  |
|  | *∆qacED1* | Quaternary ammonium  compound resistance | 155014..155454 |  |
|  | *qnrA1* | Quinolone resistance | 156672..157328 |  |
|  | *∆sul1* | Sulphonamide resistance | 159538..160159 |  |
|  | *dfrA12* | Trimethoprim resistance | 162360..162857 |  |
|  | *aadA2* | Aminoglycoside resistance | 163265..164056 |  |
|  | *qacED1* | Quaternary ammonium  compound resistance | 164220..164567 |  |
|  | *sul1* | Sulphonamide resistance | 164561..165400 |  |
|  | *ter* locus | Tellurium resistance | 245421..259615 | Plasmid backbone |
|  | *mer* locus | Mercuric resistance | 260652..264607 | ARI-A  (Tn*6384*) |
|  | *sul1* | Sulphonamide resistance | 267343..268182 |  |
|  | *qacED1* | Quaternary ammonium  compound resistance | 268176..268523 |  |
|  | *arr3* | Rifampicin resistance | 268746..269198 |  |
|  | *catB3* | Phenicol resistance | 269283..269915 |  |
|  | *bla*_OXA-1_ | Beta-lactam resistance | 270053..270883 |  |
|  | *aacA4cr* | Fluoroquinolone and aminoglycoside resistance | 271014..271613 |  |
|  | *catA2* | Phenicol resistance | 273453..274094 |  |
|  | *∆sul1* | Sulphonamide resistance | 275474..275806 |  |
|  | *qacED1* | Quaternary ammonium  compound resistance | 275800..276147 |  |
|  | *catB2* | Phenicol resistance | 276316..276948 |  |
|  | *aadA1b* | Aminoglycoside resistance | 277001..277792 |  |
|  | *aphA15* | Aminoglycoside resistance | 277909..278703 |  |
|  | *aacA4'* | Aminoglycoside resistance | 278774..279328 |  |
|  | *bla*_VIM-1_ | Beta-lactam resistance | 279436..280236 |  |
| p11219-IMP | *fosA3* | Fosfomycin resistance | 144202..144618 | ARI-B |
|  | *bla*_SHV-12_ | Beta-lactam resistance | 149288..150148 |  |
|  | *sul2* | Sulphonamide resistance | 154578..155393 |  |
|  | *strA* | Aminoglycoside resistance | 155454..156257 |  |
|  | *strB* | Aminoglycoside resistance | 156257..157093 |  |
|  | *qnrS1* | Quinolone resistance | 165003..165659 |  |
|  | *aacA4cr* | Fluoroquinolone and aminoglycoside resistance | 171841..172395 |  |
|  | *arr3* | Rifampicin resistance | 172492..172944 |  |
|  | *∆qacED1* | Quaternary ammonium  compound resistance | 173167..173223 |  |
|  | *bla*_TEM-1B_ | Beta-lactam resistance | 177157..178017 |  |
|  | *bla*_CTX-M-3_ | Beta-lactam resistance | 178799..179674 |  |
|  | *dfrA12* | Trimethoprim resistance | 181805..182302 |  |
|  | *aadA2* | Aminoglycoside resistance | 182722..183501 |  |
|  | *qacED1* | Quaternary ammonium  compound resistance | 183665..184012 |  |
|  | *sul1* | Sulphonamide resistance | 184006..184845 |  |
|  | *armA* | Aminoglycoside resistance | 187099..187872 |  |
|  | *msr(E)* | Macrolide, Lincosamide and Streptogramin B resistance | 190171..191646 |  |
|  | *mph(E)* | Macrolide resistance | 191702..192586 |  |
|  | *catA2* | Phenicol resistance | 279362..280003 | ARI-A |
|  | *ars* locus | Arsenic resistance | 281489..284636 |  |
|  | *mph(E)* | Macrolide resistance | 294791..295675 |  |
|  | *msr(E)* | Macrolide, Lincosamide and Streptogramin B resistance | 295731..297206 |  |
|  | *armA* | Aminoglycoside resistance | 299505..300278 |  |
|  | *sul1* | Sulphonamide resistance | 303692..304531 |  |
|  | *∆qacED1* | Quaternary ammonium  compound resistance | 304525..304655 |  |
|  | *sul1* | Sulphonamide resistance | 307302..308141 |  |
|  | *qacED1* | Quaternary ammonium  compound resistance | 308135..308482 |  |
|  | *bla*_IMP-4_ | Beta-lactam resistance | 310594..311334 |  |
| p12208-IMP | *fosA3* | Fosfomycin resistance | 149098..149514 | ARI-B |
|  | *bla*_SHV-12_ | Beta-lactam resistance | 154184..155044 |  |
|  | *bla*_TEM-1B_ | Beta-lactam resistance | 157213..158073 |  |
|  | Δ*armA* | Aminoglycoside resistance | 161234..161451 |  |
|  | *msr(E)* | Macrolide, Lincosamide and Streptogramin B resistance | 163750..165225 |  |
|  | *mph(E)* | Macrolide resistance | 165281..166165 |  |
|  | *ter* locus | Tellurium resistance | 257249..271443 | Plasmid backbone |
|  | *mer* locus | Mercuric resistance | 272480..276456 | ARI-A  (Tn*6383*) |
|  | *aacC2* | Aminoglycoside resistance | 280698..281558 |  |
|  | *tmrB* | Tunicamycin resistance | 281571..282113 |  |
|  | *catA2* | Phenicol resistance | 287613..288254 |  |
|  | *ars* locus | Arsenic resistance | 289740..292887 |  |
|  | *mph(E)* | Macrolide resistance | 303042..303926 |  |
|  | *msr(E)* | Macrolide, Lincosamide and Streptogramin B resistance | 303982..305457 |  |
|  | *armA* | Aminoglycoside resistance | 307756..308529 |  |
|  | *sul1* | Sulphonamide resistance | 310783..311622 |  |
|  | *qacED1* | Quaternary ammonium  compound resistance | 311616..311963 |  |
|  | *bla*_IMP-4_ | Beta-lactam resistance | 314075..314815 |  |
| pKOX_R1 | *fosA3* | Fosfomycin resistance | 147479..147895 | ARI-B |
|  | *bla*_SHV-12_ | Beta-lactam resistance | 152565..153425 |  |
|  | *sul2* | Sulphonamide resistance | 157855..158670 |  |
|  | *strA* | Aminoglycoside resistance | 158775..159533 |  |
|  | *strB* | Aminoglycoside resistance | 159533..160369 |  |
|  | *qnrS1* | Quinolone resistance | 164610..165266 |  |
|  | *dfrA1b* | Trimethoprim resistance | 171652..172125 |  |
|  | *aadA5* | Aminoglycoside resistance | 172642..173430 |  |
|  | *qacED1* | Quaternary ammonium  compound resistance | 173598..173945 |  |
|  | *sul1* | Sulphonamide resistance | 173939..174778 |  |
|  | *armA* | Aminoglycoside resistance | 178123..178896 |  |
|  | *msr(E)* | Macrolide, Lincosamide and Streptogramin B resistance | 181195..182670 |  |
|  | *mph(E)* | Macrolide resistance | 182726..183610 |  |
|  | *ars* locus | Arsenic resistance | 196047..199194 |  |
|  | *catA2* | Phenicol resistance | 200680..201297 |  |
|  | *tmrB* | Tunicamycin resistance | 211633..212175 |  |
|  | *aacC2* | Aminoglycoside resistance | 212188..213048 |  |
|  | *mer* locus | Mercuric resistance | 215952..219928 |  |
|  | *ter* locus | Tellurium resistance | 220965..233902 | Plasmid backbone |
|  | *mph(E)* | Macrolide resistance | 323372..324256 | ARI-A |
|  | *msr(E)* | Macrolide, Lincosamide and Streptogramin B resistance | 324312..325787 |  |
|  | *armA* | Aminoglycoside resistance | 328086..328859 |  |
|  | *sul1* | Sulphonamide resistance | 332204..333043 |  |
|  | *qacED1* | Quaternary ammonium  compound resistance | 333037..333384 |  |
|  | *aadA2* | Aminoglycoside resistance | 333548..334339 |  |
|  | *dfrA12* | Trimethoprim resistance | 334747..335244 |  |
|  | *bla*_CTX-M-3_ | Beta-lactam resistance | 337375..338250 |  |
|  | *bla*_TEM-1B_ | Beta-lactam resistance | 339032..339891 |  |
|  | *∆qacED1* | Quaternary ammonium  compound resistance | 343827..343883 |  |
|  | *arr3* | Rifampicin resistance | 344106..344558 |  |
|  | *aacA4cr* | Fluoroquinolone and aminoglycoside resistance | 344655..345254 |  |
| p13450-IMP | *fosA3* | Fosfomycin resistance | 144201..144617 | ARI-B |
|  | *bla*_SHV-12_ | Beta-lactam resistance | 149287..150147 |  |
|  | *sul2* | Sulphonamide resistance | 154577..155392 |  |
|  | *strA* | Aminoglycoside resistance | 155498..156256 |  |
|  | *strB* | Aminoglycoside resistance | 156262..157092 |  |
|  | *qnrS1* | Quinolone resistance | 165002..165658 |  |
|  | *bla*_IMP-4_ | Beta-lactam resistance | 171701..172441 |  |
|  | *qacED1* | Quaternary ammonium  compound resistance | 174553..174900 |  |
|  | *sul1* | Sulphonamide resistance | 174894..175733 |  |
|  | *armA* | Aminoglycoside resistance | 177987..178760 |  |
|  | *msr(E)* | Macrolide, Lincosamide and Streptogramin B resistance | 181059..182534 |  |
|  | *mph(E)* | Macrolide resistance | 182590..183474 |  |
|  | *ars* locus | Arsenic resistance | 193629..196776 |  |
|  | *catA2* | Phenicol resistance | 198262..198903 |  |
|  | *tmrB* | Tunicamycin resistance | 204403..204945 |  |
|  | *aacC2* | Aminoglycoside resistance | 204958..205818 |  |
|  | *mer* locus | Mercuric resistance | 210060..214036 |  |
|  | *ter* locus | Tellurium resistance | 215073..229267 | Plasmid backbone |
|  | *mph(E)* | Macrolide resistance | 317880..318764 | ARI-A |
|  | *msr(E)* | Macrolide, Lincosamide and Streptogramin B resistance | 318820..320295 |  |
|  | *armA* | Aminoglycoside resistance | 322593..323366 |  |
|  | *sul1* | Sulphonamide resistance | 325620..326459 |  |
|  | *qacED1* | Quaternary ammonium  compound resistance | 326453..326800 |  |
|  | *aadA2* | Aminoglycoside resistance | 326964..327755 |  |
|  | *dfrA12* | Trimethoprim resistance | 328163..328660 |  |
|  | *∆bla*_TEM-1B_ | Beta-lactam resistance | 330056..330503 |  |
|  | *arr3* | Rifampicin resistance | 334718..335170 |  |
|  | *aacA4cr* | Fluoroquinolone and aminoglycoside resistance | 335267..335866 |  |
| p19051-IMP | *fosA3* | Fosfomycin resistance | 101714..102130 | ARI-B |
|  | *bla*_SHV-12_ | Beta-lactam resistance | 106800..107660 |  |
|  | *sul2* | Sulphonamide resistance | 112090..112905 |  |
|  | *strA* | Aminoglycoside resistance | 113011..113769 |  |
|  | *strB* | Aminoglycoside resistance | 113769..114605 |  |
|  | *qnrS1* | Quinolone resistance | 122515..123171 |  |
|  | *aacA4cr* | Fluoroquinolone and aminoglycoside resistance | 129308..129907 |  |
|  | *arr3* | Rifampicin resistance | 130004..130456 |  |
|  | *bla*_TEM-1B_ | Beta-lactam resistance | 134669..135529 |  |
|  | *bla*_CTX-M-3_ | Beta-lactam resistance | 136311..137186 |  |
|  | *dfrA12* | Trimethoprim resistance | 139309..139887 |  |
|  | *aadA2* | Aminoglycoside resistance | 140213..141004 |  |
|  | *qacED1* | Quaternary ammonium  compound resistance | 141168..141515 |  |
|  | *sul1* | Sulphonamide resistance | 141509..142348 |  |
|  | *armA* | Aminoglycoside resistance | 145762..146535 |  |
|  | *msr(E)* | Macrolide, Lincosamide and Streptogramin B resistance | 148834..150309 |  |
|  | *mph(E)* | Macrolide resistance | 150365..151249 |  |
|  | *ars* locus | Arsenic resistance | 161404..164551 |  |
|  | *catA2* | Phenicol resistance | 166037..166678 |  |
|  | *tmrB* | Tunicamycin resistance | 172178..172720 |  |
|  | *aacC2* | Aminoglycoside resistance | 172733..173593 |  |
|  | *mer* locus | Mercuric resistance | 177835..181811 |  |
|  | *ter* locus | Tellurium resistance | 182848..197042 | Plasmid backbone |
|  | *mph(E)* | Macrolide resistance | 290769..291653 | ARI-A |
|  | *msr(E)* | Macrolide, Lincosamide and Streptogramin B resistance | 291709..293184 |  |
|  | *armA* | Aminoglycoside resistance | 295545..296318 |  |
|  | *sul1* | Sulphonamide resistance | 298572..299411 |  |
|  | *qnrB52* | Quinolone resistance | 299902..300546 |  |
|  | *sul1* | Sulphonamide resistance | 304293..305132 |  |
|  | *qacED1* | Quaternary ammonium  compound resistance | 305126..305473 |  |
|  | *bla*_IMP-4_ | Beta-lactam resistance | 307585..308325 |  |
