# Supplementary material for: Sequencing and Genomic Diversity Analysis of IncHI5 Plasmids
Source: Front Microbiol. 2019 Jan 14;9:3318. doi: 10.3389/fmicb.2018.03318 (PMC6339943; doi:10.3389/fmicb.2018.03318)
Supplement: TABLE S3 — Conjugation transfer features of IncHI5 plasmids analyzed. [file Table_3.docx]

**TABLE S3| Conjugation transfer features of IncHI5 plasmids analyzed**

| **Plasmid** | ***tra1* region** | ***tra2* region** | **Putative conjugation transfer ability** |
| --- | --- | --- | --- |
| p11219-IMP | Complete | Complete | Positive |
| p13450-IMP | Complete | Complete | Positive |
| pYNKP001-dfrA | Complete | Complete | Positive |
| pA324-IMP | Insertion of IS*4*-related region between *tivF3* and *∆orf3351* | Complete | Positive |
| pKP04VIM | Insertion of IS*4*-related region between *tivF3* and *∆orf3351* | Complete | Positive |
| p13190-VIM | Insertion of IS*4*-related region between *tivF3* and *∆orf3351* | Complete | Positive |
| pA708-IMP | Insertion of IS*Kox1* between *tivF*3 and *orf171* | Complete | Positive |
| p12208-IMP | Complete | Insertion of IS*Ec33* between *htdA* and *trhO* | Positive |
| pKOX_R1 | Complete | Insertion of IS*102* between *htdA* and *trhO* | Positive |
| pKpNDM1 | Insertion of IS*4*-related region between *tivF3* and *∆orf3351* | Insertion of IS*Ec33* within *tivF1* | Negative |
| p19051-IMP | Complete | Lost | Negative |
